# Unifying ‘the’ Precautionary Principle? Justification and Reflective Equilibrium

**DOI:** 10.1007/s11406-022-00582-0

**Published:** 2022-09-28

**Authors:** Tanja Rechnitzer

**Affiliations:** Leibniz Universität Hannover, Hannover, Germany

**Keywords:** Precautionary Principle, Reflective Equilibrium, Empty-Label Challenge, Justification, Conceptual Re-Engineering, Unification, Pluralism

## Abstract

The precautionary principle (PP) is an influential principle for making decisions when facing uncertain, but potentially severe, harm. However, there is a persistent disagreement about what the principle entails, exactly. It exists in a multitude of formulations and has potentially conflicting ideas associated with it. Is there even such a thing as ‘the precautionary principle’? This paper analyses the debate between unificationists and pluralists about ‘the PP’, arguing that the debate is hindered by neglecting the question of justification. It introduces reflective equilibrium as a method of justification, and sketches how it could be applied to justify a PP.

## Introduction

The basic idea of the precautionary principle (PP) is often summarized as “better safe than sorry”, meaning that we have to act to prevent harm even if we are uncertain about the likelihood or extent of possible harm. This paper addresses one of the major challenges the PP faces: The persistent disagreement about how it should be spelled out and justified as a normative principle. There is a multiplicity of interpretations, which often have apparently conflicting ideas associated with them (Rechnitzer, 2020). For example, both the minimax regret rule as well as the maximin rule have been suggested as interpretations of the PP, but these rules can lead to conflicting recommendations in the same case (Hansson, 1997).

This diversity has led some to reject the idea that there is even such a thing as *the* precautionary principle. For example, Jordan & O’Riordan (1999: 16) interpret ‘the PP’ as a “repository for a jumble of adventurous beliefs that challenge the status quo of political power, ideology, and environmental rights”. Such a repository is not necessarily without (political) force. The label ‘the precautionary principle’ could be used, e.g., to express a shared commitment to take environmental concerns more seriously. But it clearly could not serve as an action-guiding principle for decision makers (Steel, 2015: 5): If ‘the PP’ refers simultaneously to several potentially conflicting decision rules, then it is not clear what ‘the PP’ demands of us in a given situation. If we cannot say what ‘the PP’ refers to—and what not—then the term becomes little more than an empty label that can be applied to almost anything. This would make calls to follow ‘the PP’ vacuous (Steel, 2015: 6). In addition, it has been argued that the PP cannot be made more precise without sacrificing its plausibility, that is, that its appeal ultimately depends on its vacuousness (Turner & Hartzell, 2004) and that it is, in principle, incoherent (Peterson, 2006). In the remainder of this paper, I refer to this twofold challenge as the *empty label challenge*.

### Empty Label Challenge

(i) The expression ‘the precautionary principle’ refers to a repository of incoherent and potentially conflicting ideas and decision rules. (ii) It is not possible to obtain a coherent normative precautionary principle from this repository that can be justified.

We can identify two main lines of answers from proponents of precaution. Authors that I call **unificationists** argue that we can identify a core, dimensions, or substantial features, of *the* PP (e.g., Sandin 1999; Steel, 2015). This core can be used to distinguish between principles and decision rules that are versions of *the* PP, and those that are not. Once we have such a consistent, unified account of what the PP is, we can assess whether, and on what grounds, it can be justified and defended.

But others, which I call **pluralists**, argue that the unificationist project is doomed to fail because there is no core of ‘the PP’ that could unify the different interpretations (Hartzell-Nichols, 2013; Sandin & Peterson, 2019; Thalos, 2012). Instead, they argue that there are several precautionary *principles*, which cannot be unified. Because these PPs express different normative claims, they can potentially lead to conflicting recommendations. However, in themselves, they would be consistent principles with clear conditions of application and clear recommendations—which, according to pluralists, is something that cannot be achieved by attempting an unificationist approach.

In this paper, I argue that both unificationists and pluralists fail to give a satisfying answer to the empty label challenge because they fail to give a satisfying answer to the question of justification. Instead, I propose that using the method of reflective equilibrium (RE) provides the ideal tools to answer the empty label challenge and to resolve the confusion about the (dis-)unity of ‘the PP’.^1^

I analyse the positions of unificationists and pluralists in Sects. 2 and 3. Both sides seem to treat the empty label challenge as involving two sequential questions: First, what the (or a) PP *is*, and second, how it can be *justified*. I argue that by focusing on the first aspect and discussing whether or not there is a unified account, they fail to give a satisfying answer to the question of justification.

In Sect. 4, I introduce reflective equilibrium (RE) as a method which addresses both formulation and justification of a principle: Justification via RE is a *constructive* and goal-oriented process. Additionally, it often includes elements of “conceptual re-engineering”, meaning the intentional replacement of a concept in use through a newly developed concept (Brun, 2022). In Sect. 5, I sketch how one could apply RE to “re-engineer” a justified PP. In doing so, I show how some of the ideas of pluralists and unificationists can be reinterpreted in the framework of RE.

In Sect. 6, I discuss my results before drawing a conclusion in Sect. 7. While this paper does not answer which PP is justified, it shows how this question can be tackled.^2^ It thereby advances the debate by dissolving the empty label challenge through showing that it rests on the misconception that we first have to describe ‘the PP’ before being able to justify it.

## Unificationists: The PP and its Many Versions

“Unificationists”, as I call them, try to look behind the differences in formulation in order to identify a substantial core that can unify our talk of ‘the PP’. They typically do not attempt a definition in terms of necessary and sufficient conditions, but try to “distil” central features that are shared by all versions of *the* PP.

In this section, I defend an interpretation of unificationist approaches as three instances of variations of *prototype*-conceptions: Paradigm examples, substantial features, and a schema (see Murphy 2002: Chap. 3, for an overview of these positions). I argue that none of the prototype accounts does allow us to distinguish *justified* versions of the PP from those that are not, which is why they cannot answer the second part of the empty label challenge. I conclude the discussion of unificationist positions in this section with Steel (2015), who makes a significant step forward by including criteria for distinguishing sound PP versions from those that are not.

***Paradigm examples.*** Often, principle 15 of the Rio Declaration on Environment and Development, and the formulation of ‘the PP’ that resulted from the Wingspread Conference on the Precautionary Principle, are named as paradigm examples of the precautionary principle:

**Rio PP** In order to protect the environment, the precautionary approach shall be widely applied by states according to their capabilities. Where there are threats of serious or irreversible damage, lack of full scientific certainty shall not be used as a reason for postponing cost-effective measures to prevent environmental degradation. (United Nations Conference on Environment and Development 1992)

**Wingspread PP** When an activity raises threats of harm to human health or the environment, precautionary measures should be taken even if some cause and effect relationships are not fully established scientifically. (Science & Environmental Health Network (SEHN) 1998)

Insofar as these two examples are seen as constituting the core of ‘the PP’, it makes sense to see them as part of a prototype-interpretation. Prototypes are one or more “best example” of a category, and other members of the category can be arranged based on how similar they are to the prototype(s), i.e., how typical they are for the category (Rosch, 1975). Thus, the more similar a proposed PP interpretation is to one of the two prototypes, the clearer it is that it is a version of *the* PP, whereas atypical or borderline PP-versions will be further away from the prototypes.

***Substantive Features.*** The prototype-idea has also been spelled out in the form of substantive features that are important for a category (Murphy, 2002: 42–43). Thus, when unificationists name central themes that constitute the core of *the* PP, we can understand this as another prototype-interpretation. For example, Ahteensuu (2008: 37–38) claims that PP implies (A1) the anticipation of severe environmental damage and health hazards, (A2) a norm to take pre-emptive actions instead of a reactive approach which only states the obligation to remedy or compensate damage after it takes place, and (A3) that the adequate role of scientific knowledge in the environmental and health decision-making has to be redefined. However, there is no agreement on these substantial features, either: Tickner (2001: 113) includes (T1) action in the face of uncertainty, (T2) placing the burden of proof on proponents of potentially harmful activities, (T3) assessment of alternatives, and (T4) democratic decision-making structures (see also Arcuri 2007: 32–34 for again slightly different features).

***A Unifying Schema.*** The idea that all PP versions share the same underlying structure behind their superficial differences in formulation goes back to Sandin (1999) and Manson (2002) and has been taken up by a number of authors, e.g., Gardiner (2006); Ahteensuu (2008); or Steel (2013, 2015). Typically, it is identified as a tripartite structure, consisting of trigger conditions, namely (i) a damage condition and (ii) a knowledge condition, and (iii) a precautionary response. (iii) is triggered by (i) and (ii) and can range from very specific actions like bans of suspicious substances to broader approaches like implementing precautionary methodologies in risk assessment (Ahteensuu, 2008: 43). Differences between PP versions result from different substitutions for the three elements.

All these accounts might show us a way in which we can clarify what ‘the PP’ refers to. However, they cannot answer how we could justify such a principle. Why should we adhere to a principle just because it is a version of *the* PP? For example, the three elements of the schema can be assigned in very different, including very implausible or incoherent, ways, like “Whenever (1) the slightest harm (2) is logically conceivable, (3) we should prohibit any action that could cause this harm.”

Thus, prototype accounts cannot fully alleviate the concerns behind the empty label challenge: ‘The PP’ can still refer to a repository of inconsistent rules without telling us which of these are justified (Steel, 2015: 6).

***Three Unifying Core Themes***. Steel argues strongly for the need of a unified account, but one that moves beyond those that I identified as prototype-accounts. He proposes a PP interpretation which consists of three interrelated core themes (Steel, 2015: 9–11):

1. The Meta PP, which asserts that uncertainty is not a reason for inaction.
2. The “Tripod”, i.e., the schema of knowledge condition, harm condition, and recommended precaution.
3. Proportionality-Constraints, namely (i) Consistency and (ii) Efficiency.

The central idea is that one obtains versions of the PP by specifying the Tripod with respect to the demands of the Meta PP and proportionality. The Meta PP expresses the idea from the Rio PP that uncertainty is not a *reason* for inaction: While it does not itself recommend direct action, it puts procedural constraints on how harms should be assessed and how our knowledge conditions should be conceptualized; namely, the knowledge and harm condition have to be specified in such a way that uncertainty does not paralyse the decision process. The proportionality constraints ensure that a PP version is specified in a way that allow it to consistently and clearly recommend cost-efficient precautionary action: Consistency requires that a precautionary measure is not recommended against by the same PP version that is used to defend it, that is, otherwise the tripod has to be adjusted. Efficiency states that if more than one measure can be consistently recommended, then the least costly one should be chosen. To obtain a PP version, Steel (2015: 30) proposes the following strategy: (1) select a desired safety target and define the harm condition as a failure to meet this target, (2) select the least stringent knowledge condition that results in a consistently applicable version of PP given the harm condition. Throughout his book, Steel goes on to demonstrate how this interpretation can either incorporate elements of the ‘PP repository’ or can provide a reasoned basis for rejecting them.

However, as we will see in the next section, pluralists are not satisfied with such an account and argue that there is no core to ‘the PP’ that would allow to unify all valid considerations that are associated with the use of the term.

## Pluralists: Precautionary *Principles*, Not *the* PP

According to pluralists, there is nothing interesting and substantial that all PPs have in common. In this section, I present their arguments against a “core” of precaution, followed by their defence of the need for distinct precautionary principles of limited scope.

***No Essential Core***. Thalos (2012) argues that precaution can be conceptualized and translated into action in different ways that are all important and meaningful. But she denies that they do have any substantial overlap that would allow to identify an essential core that could constitute the basis for different versions of the same principle. She argues that the “pre” in precaution can be understood in different ways that emphasize how caution should be prior to something. She distinguishes (a) taking precautions under uncertainty, before all the facts are in, (b) a priority for specific values, that could refer to a strict lexical ordering of our values (e.g., something like “environmental concerns always come first”), and (c) precaution in the sense of planning and preparing ahead for an unknown future, e.g., in the special case of (d) planning ahead in order to avoid moral injustice.

Given these different categories of precaution, Thalos (2012: 174) states that it is unreasonable to think that there could be a “core” thought or principle that captures all these different concerns for precaution. She stresses that precaution is highly context-dependent, e.g., the difference between what she calls front-loading situations where we cannot intervene anymore once the process has been started, and coordinative plan situations, where we can continuously use new information and re-evaluate our actions. Similarly, Hartzell-Nichols (2013; 2017) argues that precaution can demand very different things of us based on the kinds of threat it refers to and the normative obligations underlying it, and that these aspects are too diverse to be captured by one single principle.

***Distinct Principles with Limited Scope***. As an answer to this lack of a unifying core of ‘the PP’, Hartzell-Nichols (2017) argues that we need to formulate and defend a range of *pro tanto*^3^ precautionary principles in order to adequately integrate precaution into decision-making. These PPs should have a limited scope and express different kinds of normative obligations, and could therefore conflict with each other. However, each of them would have the advantage of being precise and leading to consistent and determinant recommendations for a distinct set of circumstances. According to Hartzell-Nichols, this approach avoids many of the standard-objections against ‘the PP’:

Limiting the conditions of application to specific kinds of threat intends to avoid the paralysis-objection, one of the standard objections against ‘the PP’. According to this objection, strong versions of ‘the PP’—i.e., versions that are not too weak to provide any actual constraints on decisions—lead to paralysis: We can’t take precautionary measures against all threats, as every measure introduces itself at least a small threat of harm, which again would call for precaution, and so on (Sunstein, 2005).

Requiring that a PP expresses a distinct normative *pro tanto* obligation intends to avoid that a PP cannot provide clear guidance because of value conflicts. Hartzell-Nichols (2017: Chap. 1.3) argues that it is a problem that both environmental protection and the protection of human health are associated with ‘the PP’:

“Protecting the environment for its own sake and protecting human health are substantially different ends that will sometimes require very different things of us such that a principle that requires us to protect both will sometimes require the impossible of us.” (Hartzell-Nichols, 2013: 313–314).

Thus, in order to justify a PP, we have to identify what kind of “ought” the principle is supposed to express (Hartzell-Nichols, 2013: 315): It could, e.g., be a moral, epistemic, or prudential principle; and a moral principle might be based on specific ethical positions like anthropocentric or eco-centric ethics. Consequently, different PPs might get in conflict with each other, or with other (moral, legal, rational, …) principles. However, Hartzell-Nichols argues that this is not a weakness, but part of being a principle: The PPs express *pro tanto* obligations, and in practice, principles often have to be weighed against each other (see also Randall 2011: 97). Clearly identifying the underlying normative assumptions is supposed to help us to decide what to do in such cases (Hartzell-Nichols, 2013: 315).

***What makes a principle a*precautionary*principle?*** To answer the empty label challenge, even pluralists have to answer the question of what makes a principle a *precautionary* principle. In a recent article, Sandin & Peterson (2019: 2) advocate for a family resemblance view as an answer to the worry that without a unifying core, the term ‘the PP’ would become an empty label.^4^ According to this view, what makes a principle a PP is that it has some characteristics in common with some members of the “family of PPs”, but not necessarily with all of them—“so that *x* resembles *y* and *y* resembles *z* and so on, but without there being any resemblance between, say, *x* and *z*” (Sandin & Peterson, 2019: 2).

However, nothing ensures that only justified principles are members of the family. Thus, the family of PPs can still be identical with a repository of incoherent and potentially conflicting ideas and decision rules, and we are still lacking criteria to identify those PPs that we should adhere to. The family resemblance view thus fails to restrict the use of the term ‘the PP’ in a sufficient way to fully answer the empty label challenge.

***Discussion: Unificationists vs. Pluralists.*** Do the shortcomings of pluralist positions speak in favour of pursuing an unificationist account? That is, do we need a unified core, after all, in order to fix the referent of the term ‘the PP’ to a coherent PP that can be justified? For example, Steel argues that not all of the considerations named by pluralists are actually legitimate elements of *the* PP. He claims that his account can accommodate the notions (a), (c), and (d) named by Thalos (2012), and rejects (b), a priority for specific values, as implausible (Steel, 2015: 47–48).

Pluralists could object that Steel’s account is not really unifying *the* PP, but just developing *one* particular PP among other PPs. They might argue that while Steel is rightly narrowing down his PP by excluding some aspects, those should be taken seriously by another PP. For example, Hartzell-Nichols sees PPs as substantial normative principles, each of them expressing a distinctive normative obligation like protecting the environment, protecting human health, or avoiding catastrophic harm. She thus sees it as a “major weakness” of Steel’s approach that it leaves the value-judgments that have to be made in defining the harm condition of his PP to public deliberation (Hartzell-Nichols, 2017: 31).

But Steel would probably answer that such questions are external to PP, and have to be answered by other principles or theories prior to the application of a PP version, depending on the regulatory context and the underlying values. He aims at formulating a reasonable framework for decision-making that is not susceptible to paralysis by scientific uncertainty (Steel, 2015: 211). Thus, he sees the PP in analogy to other tools like cost-benefit analysis, which also does not define in itself what a “benefit” is.

Consequently, the debate between unificationists and pluralists could so far not resolve the disagreement about what kind of principle, or principles, ‘the PP’ is, and how many justified PPs there are. How can we move forward to address the empty label challenge? I argue that the fundamental problem with both approaches is that they start from the question what the PP *is*, without addressing how such a principle can be *justified*.

In the next section, I introduce reflective equilibrium (RE) as a fruitful method to reframe the issues about the (dis-)unity of ‘the PP’ and the justification of such a principle (or set of principles): As a method for re-engineering ‘the PP’ in a way that aims at formulating and justifying a PP which is guided by existing commitments about what ‘the PP’ is and what it demands, while at the same time aiming to (at least partially) replace those commitments. This makes it a suitable method to address both aspects of the empty label challenge at once.

## The Method of Reflective Equilibrium

Reflective equilibrium (RE) is an influential method of justification, best known from debates about moral principles. The idea goes back to Goodman’s (1983) discussion of logical validity, while Rawls (1971) coined the term “reflective equilibrium” and popularised it as a method in practical philosophy. In this paper, I propose to understand RE as a method of conceptual re-engineering, building on the works of Baumberger & Brun (2017; 2020), Brun (2013; 2020; 2022), and Elgin (1996; 2017).^5^ In the following, I focus on two features of this RE conception^6^ that make it a particularly suitable method to resolve the confusion about the (dis-)unity of ‘the PP’: Firstly, justification via RE is goal-relative, and secondly, it is constructive.

Justification via RE starts from the existing commitments about a subject matter, but aims to adjust these commitments in order to better meet certain objectives, e.g., to be able to make justified decisions. For this purpose, an epistemic agent (which can be a single individual or a group of people) searches for systematic principles that can account for their commitments while allowing them to infer, e.g., judgments on new cases. In situations of conflict between commitments and systematic principles, both sides are open to revision, until a coherent position is reached.

The difference between commitments and systematic principles is a functional difference, not one of content or of form (Brun, 2020). Commitments can include case-based, particular judgments, but also general judgments (Brun, 2013: 240; Rawls 1974: 289), e.g., “lying is wrong”. Initial commitments can also include categorizations or definitions that are important for the subject matter, e.g., a definition of what it means to lie. They constrain what we are talking about, and describe what we want to have a justified account of. These initial commitments are typically unsystematic, incomplete, and inconsistent (Elgin, 1996: 106).

This is why we search for *systematic* principles, e.g., principles that identify relevant features of the commitments. It is possible that these principles are more or less restatements of commitments (cf. Knight, 2017: 51–52), for example, if I have the commitment that it is wrong to lie, I might adopt the principle “one should not lie”. If I now try to account for my commitments with this principle, I realize that it conflicts with my commitment “you should not tell a murderer where their intended victim is, even if you know it”. I then have to decide whether I should adjust the principle, or the conflicting commitment. Referring to background theories enables argumentation for or against different ways to resolve such conflicts (Daniels, 1979).

In order to be systematic, principles have to do justice to theoretical virtues: Having theoretical virtues like accuracy, a broad scope, simplicity, or fruitfulness, is what allows them to order and systematize our commitments. It is also what will enable them to meet our pragmatic-epistemic objective that motivated us to start a justificatory process in the first place, i.e., what we want to know or understand about the subject matter and what we want to use this understanding for (Baumberger & Brun, 2017, 2020).

Which configuration of virtues is relevant depends on this overall objective. For example, if our objective were to develop a regional weather model that makes exact predictions for mountain valleys, precision would likely be more important than if we wanted to develop a climate model for the purpose of understanding the basic mechanism of global climate change. In the latter case, however, simplicity and scope might be more important.

In this way, justification is goal-relative. It is also constructive, as formulating the target system (a principle, or set of principles, or a theory, model, etc.) is part of the process of justification.^7^ The target system is constructed with respect to our initial commitments on the one hand, and our pragmatic-epistemic goals on the other hand. Through this process, our commitments are revised and systematized, rendering them more coherent. Thus, we have to distinguish the two levels of (i) the initial commitments from which the process of justification starts, and (ii) the resulting commitments, which ideally will be justified through being in agreement with a (set of) systematic principle(s).

However, not just any adjustment to the initial commitments is admissible. As I said above, our initial commitments constrain the subject matter—even if incomplete and inconsistent, they describe what it is that we want to have a justified account of. Thus, we need to be able to show how the resulting equilibrium can be obtained in a reasoned way from the initial commitments (Elgin, 2017: 66). Looking back, we need to be able to give a plausible argument for every adjustment that was made to an initial commitment. This ensures that, while adjusting commitments and correcting false assumptions and biases, we do not implement arbitrary changes or even change the subject. By constraining which adjustments are admissible, the initial commitments thereby also constrain and inform our choice of principle(s): Commitments are adjusted with respect to systematic principles while having to respect initial commitments, and systematic principles are formulated and adjusted with respect to the commitments and our pragmatic-epistemic objective.

Thus, a principle is justified via RE if (a) it is in agreement with the resulting commitments, (b) the resulting commitments respect the initial commitments, (c) the principle can be supported by independent background theories, (d) the principle has theoretical virtues that allow it to meet the pragmatic-epistemic objective, and (e) adopting the principle makes the resulting position at least as plausible as if relevant alternative principles were adopted.

In the next section, I sketch how one could apply this RE conception as a method of justification to the debate about the unity or plurality of ‘the PP’.

## Locating the (Dis)Unity-Debate in the Framework of RE

If someone wanted to use RE to justify a PP, how should they approach this? And would they end up with a unified account of *the* PP, or with several PPs? In this section, I sketch the answer to the first question. I also argue that there is no predefined answer to the second question—but that this is no obstacle for addressing the empty label challenge.

In particular, I make three main points: First, the empty label challenge is only a problem as long as we get stuck at focusing on the level of the initial commitments. Once we recognize that commitments need to be adjusted and revised in the process of justification, the empty label challenge turns out to be merely the starting point of a justificatory process, not a fundamental challenge to it. Consequently, it is irrelevant whether or not we can identify a unifying core of ‘the PP’ at the level of the initial commitments. Second, unificationists like Steel are correct that to be justified, a principle needs to be able to systematize the resulting commitments and to relate them to each other as part of a coherent—in other words, unified—account. Third, however, because justification via RE is goal-relative, pluralists like Hartzell-Nichols are correct that it is possible that more than one PP can be justified, even if the process were to start from the same set of initial commitments. RE entails a sort of meta-pluralism: Justification with respect to a specific objective aims at unification, but there can be several legitimate objectives and consequently several justified PPs. That is, even if the participants in the debate were to agree on the objectives, and reach a collective unified equilibrium position, there would be nothing necessary or “essential” about this unified PP—unification on a global level is no requirement for justification. I now elaborate these points while sketching how an RE-process could be conducted.

***Initial Commitments.*** An application of RE would start from relevant initial commitments on precaution and ‘the PP’. By clarifying and making explicit what people usually refer to, or imply, when talking about ‘the PP’, and which judgments and values they endorse in this context, we can identify a shared pre-systematic^8^ starting point. The initial commitments provisionally delineate the subject matter, but it is to be expected that they are disconnected, inconsistent, and incomplete, and that they include ambiguous or contested concepts. For example, we might be committed to the judgment that measures to prevent harm from asbestos dust should have been taken earlier (see Harremoës et al., 2001). But we might be unsure how much earlier, and how much indication of harm should be required for the regulation of other substances. We might think that the maximin-rule is a good expression of precaution, while also thinking that the Rio Declaration is an important formulation of ‘the PP’, and being unsure how the two relate to each other. We might be committed that precautions against catastrophic climate change harms need to be taken urgently, without knowing whether researching climate engineering technologies should count as such a precaution. And so on.

As these examples show, the initial commitments include particular judgments on relevant cases, but also general commitments, and commitments to definitions or categorizations. In fact, I argue that many of the unificationist candidates for substantial themes of the PP should be better seen as initial commitments. For example, that the Rio or the Wingspread PP are often named as paradigmatic PP examples indicates at least some commitment to seeing them as expressions of precautionary thinking that should be respected. “Respect” in RE does not mean that they are save from revision. But as part of the initial commitments, they partly determine which system is chosen, and even if they ultimately were rejected, another system might result than based on another set of input commitments.

The same holds for what are named as “substantial themes” of the PP like the belief that the burden of proof should be placed on proponents of an action, or that we should be especially precautious with respect to the environment, etc. If we see them as initial commitments, the lack of consensus about these “themes” ceases to be a problem.

It is indeed very likely that there will be no “consistent core” or set of “substantial themes” that all PP theorists agree on. But this is not, as the empty label challenge makes it seem, an insurmountable problem for the justification of a PP. Initial commitments provide a shared starting point—where a PP theorist can, e.g., agree that concern for the environment is often associated with ‘the PP’, even if they themselves think that a sound PP should not be restricted in such a way. Initial commitments thus inform and constrain the development of a target principle, but not in the form of substantial and fixed statements about a core of ‘the PP’. It is not problematic if our shared starting point for what ‘the PP’ refers to is a “repository of incoherent and potentially conflicting ideas and decision rules”. Whether the initial commitments can be adjusted in order to obtain a coherent account that meets specific objectives is something that will be a result, not a precondition, of the justificatory process. Requiring that we first identify a consistent PP interpretation would, in fact, threaten to severely limit the process by excluding potentially relevant commitments.

At the level of our initial commitments, the empty label challenge poses no threat. If we think it does, then we are overlooking the constructive aspect of justification. However, the question remains whether we can arrive at a coherent, justified interpretation of ‘the PP’ (or several such PPs).

***Pragmatic-Epistemic Objective.*** As explained above, justification via RE is relative to our pragmatic-epistemic objectives. There is often not one overall best option to resolve trade-offs, and then we have to decide what best serves our objective. For example, one principle candidate might allow to infer more precise verdicts, while another is applicable to a broader range of cases. Which one we should choose will depend on what we want to learn about the subject matter. In the context of ‘the PP’, possible objectives are, e.g., justifying a formal decision rule for decisions under uncertainty, or a set of epistemic principles for weighing evidence in situations of uncertain harm, or a principle that tells us what the morally right way to deal with long-term, low-probability threats of catastrophic harm.

***Systematic Principles.*** To introduce candidates for systematic principles, we can either formulate completely new candidates, or draw on our commitments as well as existing proposals from the literature. For example, we might assess how well paradigmatic PP examples such as the Wingspread PP can fulfil the role of a systematic principle. The PP-schema, i.e., the tripartite structure of harm condition, knowledge condition, and precautionary measure, can also be a first step towards systematizing commitments. We could, like Steel does, try to find criteria for how the three elements should be adjusted with respect to each other in order to lead to judgments that are in agreement with our commitments.

Because the difference between commitments and systematic principles is a functional one, something can appear on both sides. For example, unificationists who think that the schema is a substantial aspect of ‘the PP’ can have a strong commitment to it. RE forces us to acknowledge, however, that no matter how strong an initial commitment is, there is never a guarantee that it will survive the process. Thus, even a strong commitment should not be regarded as an element of a fixed “core”.

***The Process of Adjustments and its Results.*** Without a core that is underlying the different ways to systematize our initial commitments, how can we decide which resulting principle is, and which is not, a *precautionary* principle? With RE, we can also answer this question: What makes a principle a *precautionary* principle is that we can plausibly argue that it is part of a position in which the adjusted commitments respect the initial commitments that we took to constrain the subject matter. That is, we have to be able to show how the resulting position can be obtained in a reasoned way from the initial commitments. Thus, ensuring that different people talking about ‘the PP’ do not merely talk past each other does not presuppose a “core” but a shared understanding of the subject matter—which can include ambiguous, vague, or inconsistent elements.

***Unified Account or Pluralism?*** At the end of the justificatory process, will we end up with an unificationist or a pluralist position? Rejecting the idea of an underlying “core” speaks against unificationist positions. Acknowledging that for different objectives, different justified PPs might result, speaks in favour of a pluralist position.

However, by shifting the focus from the initial commitments to the resulting position, we can save part of the unificationist approach: With respect to a given objective, there is indeed a pressure to unify. Conflicts between commitments, or between commitments and systematic principles, need to be amended. We cannot justify a system that consists of several principles that can lead to conflicting recommendations. Justification via RE requires that we identify, in the form of systematic principles, what the relevant features are that allow us to categorize our commitments and to infer informative and clear-cut judgments.

Nonetheless, what unificationists overlook is that justification is goal-relative. When Steel (2015) develops his “unified” PP, this happens with respect to the goal of formulating a reasonable framework for decision-making. This leads to the exclusion of substantial value commitments, as, in such a framework for policy-making, value-judgments are left to public deliberation. Given his pragmatic-epistemic objective, Steel can make good arguments for why such considerations should be excluded.

When talking about pluralism, Hartzell-Nichols (2013; 2017) is looking at the broader picture of what is associated with PPs in various contexts, and for what kind of objectives they have been proposed. When defending her own PP interpretation, she does this with respect to a specific objective. Her goal is to defend a PP that gives direct guidance to policy-makers with respect to threats of catastrophic harm, in particular from climate change. Part of this approach is to develop a powerful argument for how, and in which ways, climate change is harmful.

I am not going to discuss here whether Steel’s or Hartzell-Nichols’ proposal is better justified. But with RE, we can explain how different PPs can result with respect to different goals. Even if they were justified to the same degree, they would be justified with respect to different objectives.

In spite of this goal-relative meta-pluralism, I argue that RE favours more unified accounts, all else being equal. Developing too many different positions, each justified with respect to a slightly different goal, is also a loss of systematicity. Having a broad scope is an important theoretical virtue for a principle: A principle that can provide guidance in more cases is certainly preferable over one that is applicable to fewer cases, all else being equal. Scope is thus a virtue that should not be traded off lightly. Ideally, we would find a unified account that reasonable meets all our objectives.

However, often not all else is being equal, and sometimes we will have to sacrifice scope in order to increase, e.g., the agreement between our commitments and the systematic principle(s). For example, we might be committed both to anthropocentric reasons for precaution as well as to eco-centric reasons, but could be unable to construct a target PP that can account for both concerns (see Hartzell-Nichols 2013: 313–314 for an example how such concerns might conflict). This would require that we revise our position, e.g., by restricting its scope to either harms to the environment or harms to human wellbeing in order to reach a position that is coherent, and in a state of reflective equilibrium.

The empty label challenge is answered, I argue, once we can demonstrate how we obtained a justified, coherent PP from our initial commitments. That our starting point is incoherent is no fundamental problem. And whether or not we end up with a unified account, or with several goal-relative justified PPs, is irrelevant for addressing the empty label challenge.

In the next section, I discuss three challenges to my approach, before drawing a conclusion in Sect. 7.

## Results and Discussion

Above, I have argued that using RE as a method of justification for PPs provides a fruitful way to address the empty label challenge. In the following, I address what I take to be the three most pressing challenges to my proposal.

First, some readers might not be convinced that RE really is the best method to use for the justification of a PP, or might even object to the use of the method in general. I cannot give a defence of RE against its critics here (but see Knight 2017; Tersman, 2018; Walden, 2013). However, I hope to have shown that it is at the very least a more fruitful way to approach the justification of a PP than unificationist or pluralist approaches.

Furthermore, RE can take into account ideas from both sides in a way that will enable us to make progress towards a justified PP: Due to RE’s pressure to systematize, and to reach a coherent position, the method favours unificationist ideas to some extent. The goal is to identify features that can systematize our commitments as best as possible. However, because our starting point is incoherent, we will have to give up some of our initial commitments, or adjust them, in order to arrive at a coherent position. And often, there will not be a uniquely best way to resolve conflicts. This is why justification via RE entails a certain meta-pluralism: Adjustments between commitments and systematic principles are partly guided by our pragmatic-epistemic objective, i.e., what we want to understand about the subject matter and what we want to use this understanding for. Consequently, with respect to different objectives, more than one justified PP can result.^9^

Second, especially unificationists might object that as long as it is possible that more than one PP results, we still do not know which of them we should accept. Thus, they might argue that my RE approach does not actually make the discussion any easier, or more fruitful.

However, RE is not only a method for the justification of individual principles. It also provides us with the means to analyse and compare different PP candidates, e.g., according to the commitments they entail, the epistemic goals they fulfil, the background they accept, and which adjustments might have been made during an RE process: How did system and commitments get weighed against each other in case of a conflict, and why? It thereby enables us to make an informed, justified choice between different PP proposals.

It seems reasonable to assume that we share some strong commitments about what is or is not a precautionary principle. Whether or not we accept a resulting system as a precautionary principle will partly depend on whether or not these commitments are adequately respected. But a meaningful discussion about PPs and their demands does neither presuppose any essentialist core of *the* PP, nor is it to be expected that each attempt of formulating and justifying a PP will end at the same resulting position.

Third, opponents of ‘the PP’ might insist that the empty label challenge has not been resolved. They might claim that our initial commitments concerning ‘the PP’ are too diverse and inconsistent to be systematized into a coherent position. However, this is a much stronger claim than pointing out that our existing commitments concerning ‘the PP’ are incoherent. As explained, the RE process can, given good reasons, involve drastic revisions of initial commitments. Of course, there is no guarantee that applying RE will lead us to a justified PP: The method itself does not guarantee that we will be successful in applying it. Nevertheless, RE forces us to shift the focus of our discussion from our initial commitments to the different ways in which we can attempt to construct and justify a systematic PP with respect to a specific pragmatic epistemic objective. Thus, even if its application does not lead to a justified PP, using RE would still advance the debate.

## Conclusion

I have argued that the perspective of reflective equilibrium (RE) allows us to dissolve the empty label challenge. As I have shown, unificationists try to answer the challenge by arguing that there is a coherent core of *the* PP. Pluralists concede that there is no consistent interpretation of ‘the PP’, but argue that we can categorize the different ideas associated with it into different, distinct, PPs which can be justified.

I have argued that both sides ultimately fail to give a satisfying answer to the empty label challenge, because they do not give a satisfying answer to the question of justification, partly because they overlook the interconnections between theory development and justification. With RE, we can acknowledge that our starting point—our initial commitments about ‘the PP’ and its implications—is incoherent, without a unifying “core”. However, this incoherence is a call to start a process of justification, not a sign that justification is impossible. Thus, we do not have to be able to give a consistent account of what ‘the PP’ is prior to being able to justify it. What makes a principle a *precautionary* principle is its tie to our initial commitments on the subject matter. What makes a PP justified is being part of a coherent position that is in reflective equilibrium, that is, the principle needs to be in agreement with a revised set of these initial commitments, while being able to meet our pragmatic-epistemic objective.

## Data Availability

Not applicable.

## Footnotes

1 I use ‘the PP’ to reference the term ‘precautionary principle’, when it is open to discussion what, if anything, it refers to. I use “*the* precautionary principle/*the* PP” to refer to the unficicationist position, and “precautionary *principles*/PPs” to refer to the pluralist position. Occasionally, I use “a PP” or “PP formulations” to refer to specific formulations, leaving open whether this is a version of “*the* PP” or one of several “PPs”.

2 See Rechnitzer (2022) for an extensive case study in which RE is applied to justify a PP.

3 In the 2013 article, Hartzell-Nichols speaks of *prima facie* principles, but in her book from 2017, she argues that they are better understood as *pro tanto* principles. I decided to follow the later terminology.

4 Interestingly, this pluralist position might be well compatible with unificationist accounts that are focusing on prototypes like paradigm examples or substantive features. In fact, the family resemblance view is typically seen as a variation of a prototype-account (see Murphy 2002: 42).

5 I think there are good reasons to see this interpretation as consistent with Rawls’s work, but exploring the relation in more detail is not within the scope of this paper.

6 For a more in-depth discussion of my understanding of RE, see Rechnitzer (2022).

7 This process can of course make use of already established candidates for theories or principles. However, their exact interpretation and specification will typically undergo at least some changes in the process.

8 Pre-systematic in the sense of previous to the current project of systematization, e.g., via RE; not meaning something like “unsystematic”.

9 This is not the only reason why more than one justified principle might result with RE, but the most important one for this debate, which is why I am not going into all the details here. See Rechnitzer (2022) for more.
